# Proteomic analysis of eleven tissues in the Chinese giant salamander (*Andrias davidianus*)

**DOI:** 10.1038/s41598-019-50909-3

**Published:** 2019-11-11

**Authors:** Xiaofang Geng, Jianlin Guo, Xiayan Zang, Cuifang Chang, Haitao Shang, Hong Wei, Cunshuan Xu

**Affiliations:** 1 State Key Laboratory Cultivation Base for Cell Differentiation Regulation, College of Life Science, Henan Normal University, Xinxiang, China; 2 Henan Key Laboratory of immunology and targeted therapy, Henan Collaborative Innovation Center of Molecular Diagnosis and Laboratory Medicine, School of Laboratory Medicine, Xinxiang Medical University, Xinxiang, China; 3 The Engineering Technology Research Center for Germ-free and Genome-editing animal, Key Laboratory of Agricultural Animal Genetics, Breeding, and Reproduction of the Ministry of Education & Key Laboratory of Swine Genetics and Breeding of Ministry of Agriculture and Rural Affairs, The Cooperative Innovation Center for Sustainable Pig Production, Huazhong Agricultural University, Wuhan, China; 4 Department of Laboratory Animal Science, College of Basic Medical Sciences, Third Military Medical University, Chongqing, China

**Keywords:** Proteomics, Animal physiology

## Abstract

The Chinese giant salamander (*Andrias davidianus*, CGS) is the largest extant amphibian species in the world. Global quantitative proteome analysis of multiple tissues would indicate tissue-specific physiological processes and clarify the function of each protein from a whole-organism perspective. This study performed proteome analysis of eleven tissues collected from adult CGSs using iTRAQ coupled with LC-MS/MS technology. Based on the predicted protein database from previously obtained CGS transcriptome data, 2153 proteins were identified for subsequent analysis. A weighted gene co-expression network analysis (WGCNA) clustered 2153 proteins into 17 co-expressed modules, which will be useful for predicting the functions of unannotated proteins. The protein levels of molecular complexes with housekeeping functions, such as ribosomes, spliceosomes and mitochondrial respiratory chain complexes, were tightly regulated in different tissues of the CGS, as they are in mammalian tissues. Transcription regulator, pathway and bio-functional analysis of tissue-specific proteins showed that highly expressed proteins largely reflected the physiological functions of specific tissues. Our data, as an initial atlas of protein expression of an amphibian species, will be useful for further molecular biology research on CGS.

## Introduction

The Chinese giant salamander (*Andrias davidianus*, CGS), endemic to Mainland China, is the largest extant amphibian species in the world. This species, considered a living fossil, may represent the transitional from aquatic to terrestrial animal life^1^. Due to habitat destruction, climate change and overhunting, the natural populations of CGS have sharply declined, leading to their inclusion in annex I of the Convention on International Trade in Endangered Species of Wild Fauna and Flora (CITES) and in class II of the national list of protected animals in China^2^. Despite their unique life-history characteristics, this species remains poorly characterized at the molecular level. Our current understanding of the long-lived CGS is restricted to the information provided by global transcriptome analyses^3–6^. Although transcriptome analysis provides valuable information at the mRNA level, it does not necessarily reflect protein expression levels accurately. Therefore, global proteome analysis of multiple tissues is extremely important for identification of molecular regulators and effectors of their biological functions.

In our previous study, we performed proteome analysis of CGS skin and *Cynops orientalis* liver using two-dimensional gel electrophoresis coupled with MALDI-TOF/MS technology^7–9^, but this approach has a major limitation in that it has only moderate reproducibility in relative peptide quantification. Currently, iTRAQ-based quantitative proteomic analysis using LC-MS/MS with very high sensitivity and specificity has been reported in amphibian *Cynops orientalis* limb regeneration and *Xenopus laevis* gonad tissue^10,11^. In a new report, a proteomic analysis of the axolotl tail was searched against an axolotl mRNA-Seq database, and the results were translated into protein sequences and annotated to process LC-MS/MS data^12^. However, proteomic analysis of CGS tissues using LC-MS/MS has not been reported. Here, we conducted a broad proteome analysis of eleven CGS tissues using iTRAQ coupled with LC-MS/MS technology, and a predicted protein database from previously obtained CGS transcriptome data^6^ was used to significantly improve the effectiveness of proteomic analysis. This study provided a proteome-scale map for different tissues in the CGS and identified some tissue-specific proteins. These data will be useful for future molecular biology studies of notable features of the CGS, such as longevity and starvation endurance.

## Results and Discussion

### Protein identification in eleven CGS tissues

To maximize information on the CGS proteome, we prepared and identified protein extracts from eleven tissues of three adult CGS using iTRAQ coupled with LC-MS/MS technology. The mass data were searched against the predicted protein database from a CGS transcriptome database previously obtained using Mascot 2.2 search engine, then quantitatively analyzed using Proteome Discoverer 1.4 software. A false discovery rate (FDR) ≤ 0.01 was utilized to filter out the data, and a total of 4607 proteins with quantitative information were identified. Among them, 2153 proteins shared by two iTRAQ 8-plex experiments were used for subsequent analysis (Table S1). The protein mass distribution was mainly concentrated at 10–100 kDa; this range contained 85.0% of the 2153 proteins. Approximately 91% of the proteins had more than one unique peptide. Approximately 73% of the proteins had sequence coverage above 10%. These data represent protein expression profiles across different tissues of CGS, and there was high overlap of the identified proteins between the various tissues.

### Western blot validation

**Figure 1:**
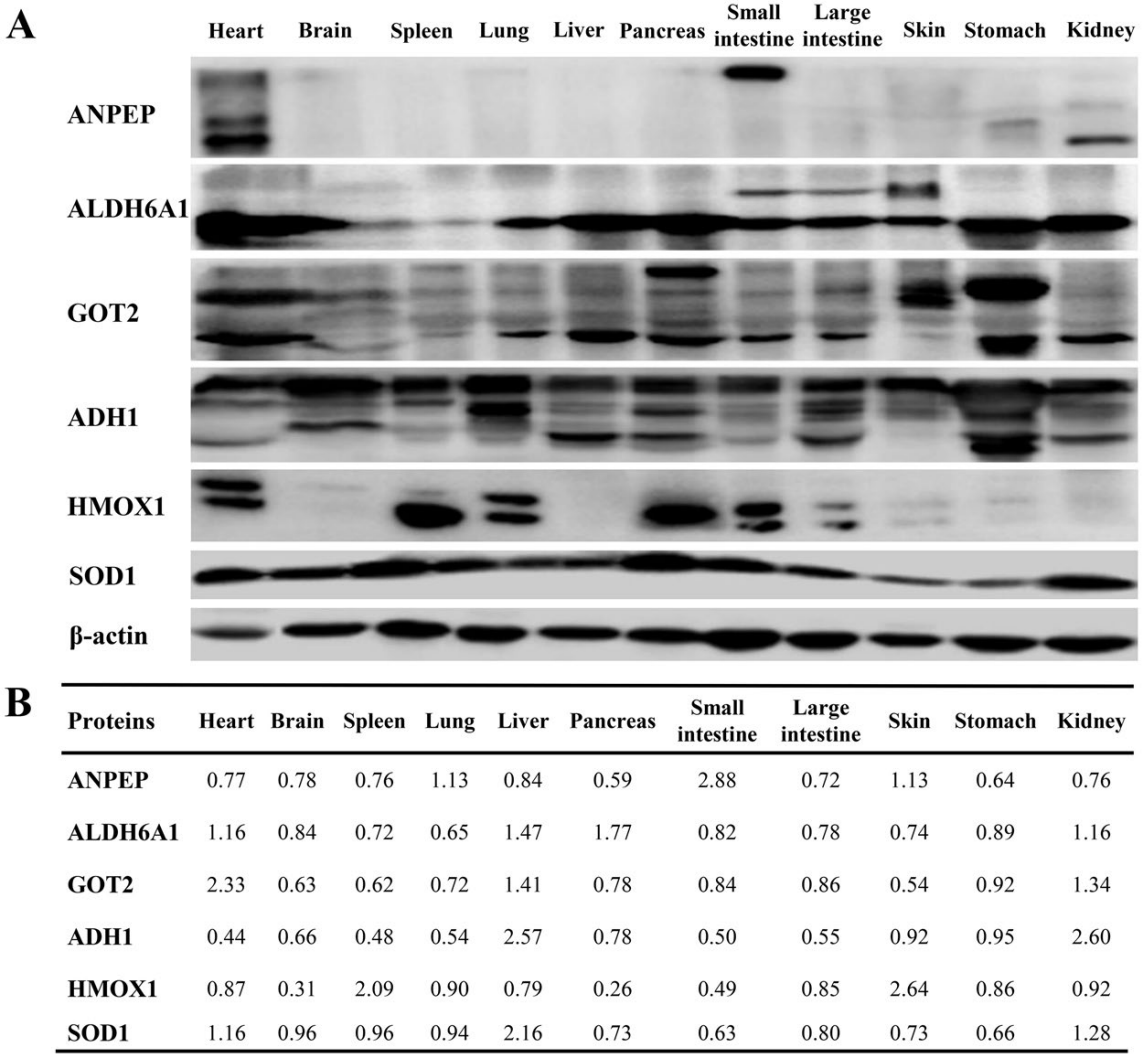
Western blot validation. (**A**) Protein expression levels were detected by Western blot. (**B**) Relative protein levels detected by iTRAQ.

Antibodies against salamander proteins were not available; therefore, mammalian antibodies were utilized to validate the reliability of protein abundance levels in different tissues of CGS by Western blot. This study randomly tested the expression levels of six proteins (ANPEP, ALDH6A1, GOT2, ADH1, HMOX1 and SOD1) in eleven CGS tissues. The results showed that ANPEP was highly expressed in the small intestine of the CGS; ALHH6A1 and GOT2 were highly expressed in the heart, liver, pancreas, stomach and kidney; ADH1 was highly expressed in the liver and stomach; HMOX1 was highly expressed in the heart, spleen, lung and pancreas; SOD1 was highly expressed in most tissues of CGS except skin and stomach (Figs 1A and S1). The protein expression trends were similar to the iTRAQ results (Fig. 1B), suggesting that the results of iTRAQ were relatively reliable.

### Tissue-specific proteins and secreted proteins of the CGS

Considering that tissue-specific proteins have elevated expression levels relative to the internal standard in only one or a few related tissues, proteins with relative expression >2 were considered to be highly expressed in specific tissues in this study. We identified 922 out of 2153 proteins as being specifically expressed in only one tissue or a few related tissues (Table S1). Among them, many mitochondrial proteins were found to be highly expressed in the heart of the CGS, which demonstrates the importance of energy metabolism for cardiac contraction. Liver-enriched proteins were mainly associated with detoxification, metabolism and glycogen storage, functions that are consistent with the key roles of the liver. Many ribosome proteins elevated in the pancreas of the CGS were involved in translation in support of secretory activity. Transcriptome analysis of human tissues and organs found many enriched genes in the brain and liver and relatively few in the lung and adipose tissue^13^. However, we found that the CGS brain had relatively few enriched proteins. This can be explained by the very low level of evolutionary specialization of the CGS brain. This demonstrates that tissue specificity depends mostly on the highly expressed proteins.

Secreted proteins are responsible for the crosstalk among cells or tissues. SignalP 4.0 was used for signal-peptide prediction, and 261 secreted proteins were identified, accounting for 12.1% of the total of 2153 proteins (Table S1). The results showed that the skin and pancreas had the largest numbers of highly expressed secreted proteins. This can be explained by the fact that amphibian skin has developed abundant mucous and granular glands^14^ and the fact that more than 70% of the transcripts from the pancreas encode secreted proteins^13^. Additionally, mucin-2 was most highly expressed in the large intestine and small intestine, consistent with the results obtained by *Velcich et al*.^15^. Mucin-2 secreted by intestinal goblet cells is the major component of the intestinal mucus layer, which forms a physical barrier between the underlying epithelium and the lumen of the gastrointestinal tract^16^.

### Functional classification of the CGS proteome

Clusters of Orthologous Groups (COGs) were used for functional classification of proteins based on the annotation results of the CGS transcriptome. Out of the 2153 proteins, 1449 proteins were assigned to COG classifications (Fig. S2A). To better understand the biological functions and interactions of these proteins, we used the Kyoto Encyclopedia of Genes and Genomes (KEGG) Pathway Database to identify biological pathways. Of the 2153 proteins, 339 proteins were assigned to metabolic pathways (Fig. S2B). Surprisingly, various proteins were involved in the AMPK, FoxO and HIF-1 signaling pathways, which regulate longevity and starvation response^17–19^. Among them, superoxide dismutase, catalase, heat shock proteins, adiponectin, and the autophagy protein ATG7 positively control longevity, while ribosomal proteins inhibit longevity^19,20^. Moreover, autophagy, stress defense mechanisms, and survival pathways are strengthened in long-lived animals, while proinflammatory mediators and cellular growth are attenuated. Starvation response involves glycolysis/gluconeogenesis, fatty acid degradation, synthesis and degradation of ketone bodies, and regulation of lipolysis in adipocytes^21^. These data provide a foundation for future research on the longevity and starvation endurance of CGS, a long-lived amphibian species.

### Clustering of tissue proteomes of CGS

**Figure 2:**
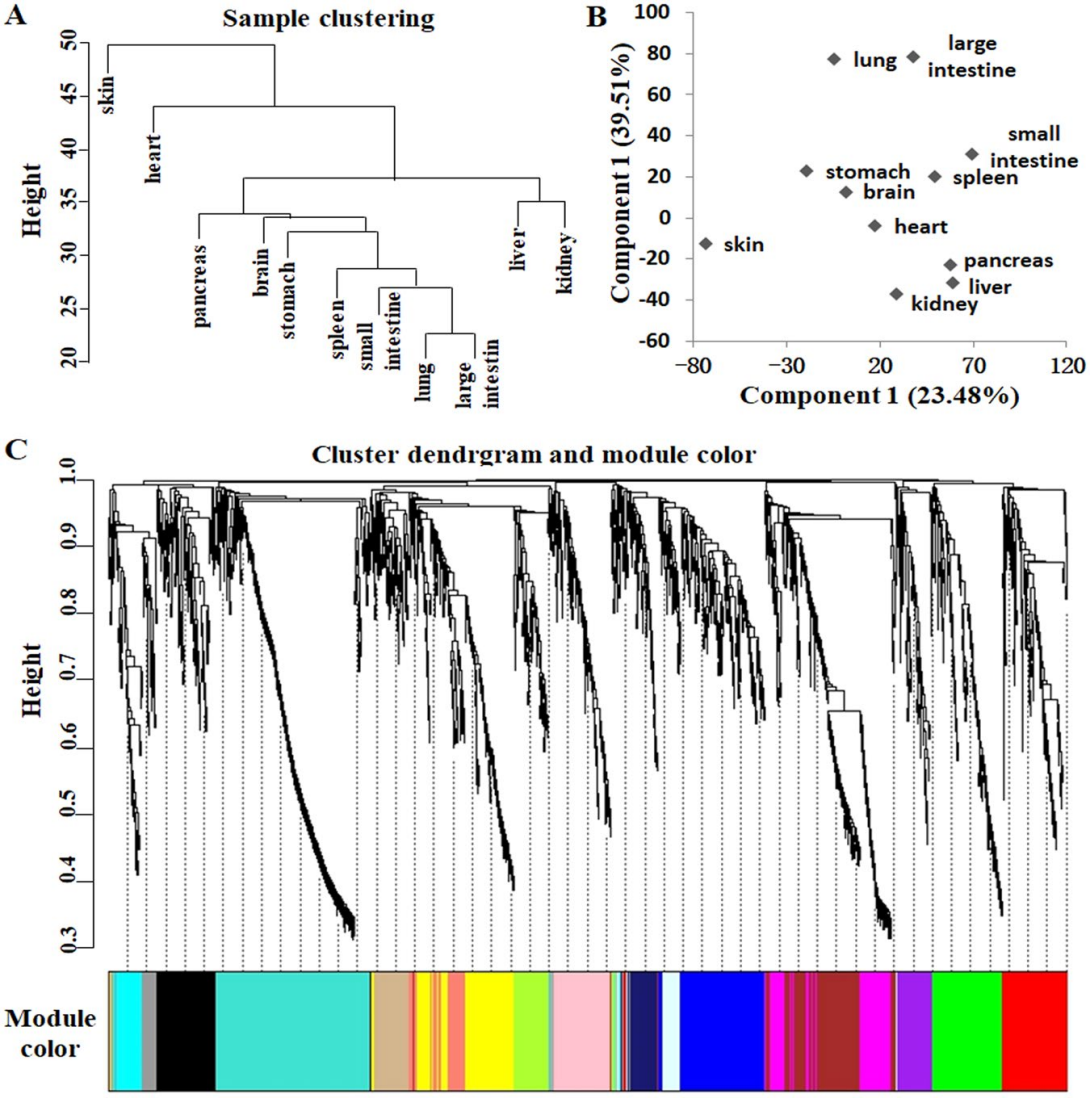
Clustering and co-expression module analysis of tissue proteomes of the Chinese giant salamander. (**A**) Hierarchical clustering of tissue proteomes. (**B**) Principal component analysis of tissue proteomes. (**C**) Co-expression module analysis of tissue proteomes.

To characterize the relationship among eleven tissues of CGS, we employed clustering and principal component analysis in this study. The results of clustering showed that the liver and kidney tightly clustered together, as did the lung, small intestine and large intestine, indicating that different tissues have related functions (Fig. 2A). The clustering analysis placed skin tissue farthest from all other tissues, corresponding to the results of principal component analysis (Fig. 2B), due to multiple unique proteins and functions of the skin.

WGCNA, which helps to identify the functions of unannotated proteins by correlating them with annotated proteins, was utilized to detect tissue-specific differences. WGCNA analysis clustered 2153 proteins into 17 coexpressed modules, which are denoted by different colors (Table S2, Fig. 2C). The correlation between the proteins and physiological functions was analyzed for enrichment of GO annotations and KEGG pathways using DAVID (Table 1). For example, the turquoise module had the largest number of co-expression proteins; these proteins were highly expressed in the pancreas of the CGS and significantly enriched for functions concerning ribosomes and translation, suggesting that this module strongly correlates with the function of pancreas tissue and that other unannotated proteins in this module may also participate in similar functions. The most prominently enriched categories for the green module were “metabolic pathways”, “drug metabolism-cytochrome P450”, “retinol metabolism” and “urea cycle”. This module strongly correlates with the physiology of liver tissue, being in accordance with key metabolic and detoxifying functions of the liver. Our data will be useful for identifying novel tissue-specific functions of known proteins and for predicting functions of unknown proteins based on the assumption that proteins in the same cluster have similar functions.

**Table 1:** Biofunction and KEGG pathways enriched in modules by DAVID analysis.

Category	Term	Count	P-value	FDR
**Turquoise (353)**				
KEGG pathway	Ribosome	60	3.78E-50	3.96E-47
Biological process	Translation	52	2.18E-39	2.94E-36
KEGG pathway	Protein processing in endoplasmic reticulum	32	1.23E-13	1.29E-10
Biological process	Formation of translation preinitiation complex	9	1.95E-09	2.63E-06
Biological process	Translational initiation	8	6.96E-08	9.39E-05
KEGG pathway	Aminoacyl-tRNA biosynthesis	12	5.98E-07	6.26E-04
**Blue (206)**				
KEGG pathway	Proteasome	11	9.22E-10	9.46E-07
KEGG pathway	Spliceosome	14	4.53E-08	4.65E-05
**Brown (181)**				
KEGG pathway	Oxidative phosphorylation	39	1.95E-33	2.06E-30
KEGG pathway	Metabolic pathways	87	2.06E-31	2.17E-28
KEGG pathway	Carbon metabolism	27	7.65E-20	8.07E-17
KEGG pathway	Citrate cycle (TCA cycle)	17	1.23E-19	1.30E-16
Biological process	ATP synthesis coupled proton transport	10	3.13E-13	3.78E-10
KEGG pathway	Fatty acid metabolism	12	7.21E-09	7.61E-06
KEGG pathway	Pyruvate metabolism	10	1.99E-07	2.10E-04
**Green (158)**				
KEGG pathway	Metabolic pathways	54	2.01E-15	2.10E-12
KEGG pathway	Tyrosine metabolism	12	1.58E-11	1.66E-08
KEGG pathway	Drug metabolism - cytochrome P450	11	3.03E-10	3.19E-07
KEGG pathway	Metabolism of xenobiotics by cytochrome P450	10	6.99E-09	7.35E-06
KEGG pathway	Biosynthesis of antibiotics	15	5.69E-06	5.99E-03
KEGG pathway	Retinol metabolism	8	1.06E-05	1.12E-02
Biological process	Urea cycle	4	1.25E-05	1.45E-02
KEGG pathway	Biosynthesis of amino acids	9	1.54E-05	1.62E-02
**Red (147)**				
KEGG pathway	Metabolic pathways	48	2.62E-09	2.79E-06
KEGG pathway	Peroxisome	12	4.55E-07	4.85E-04
KEGG pathway	Tryptophan metabolism	9	7.64E-07	8.14E-04
Biological process	Carbohydrate metabolic process	9	8.29E-06	9.88E-03
KEGG pathway	Fatty acid degradation	8	8.47E-06	9.02E-03
KEGG pathway	Lysosome	12	1.45E-05	1.54E-02
**Black (134)**				
KEGG pathway	ECM-receptor interaction	10	2.55E-08	2.38E-05
KEGG pathway	Focal adhesion	11	8.42E-06	7.83E-03
**Pink (128)**				
KEGG pathway	Phagosome	9	4.57E-05	4.63E-02
**Purple (80)**				
KEGG pathway	PPAR signaling pathway	6	2.77E-05	2.63E-02
**Cyan (72)**				
KEGG pathway	Focal adhesion	15	1.20E-12	1.03E-09
KEGG pathway	Regulation of actin cytoskeleton	10	1.64E-06	1.41E-03

### Coregulation of protein complexes

**Figure 3:**
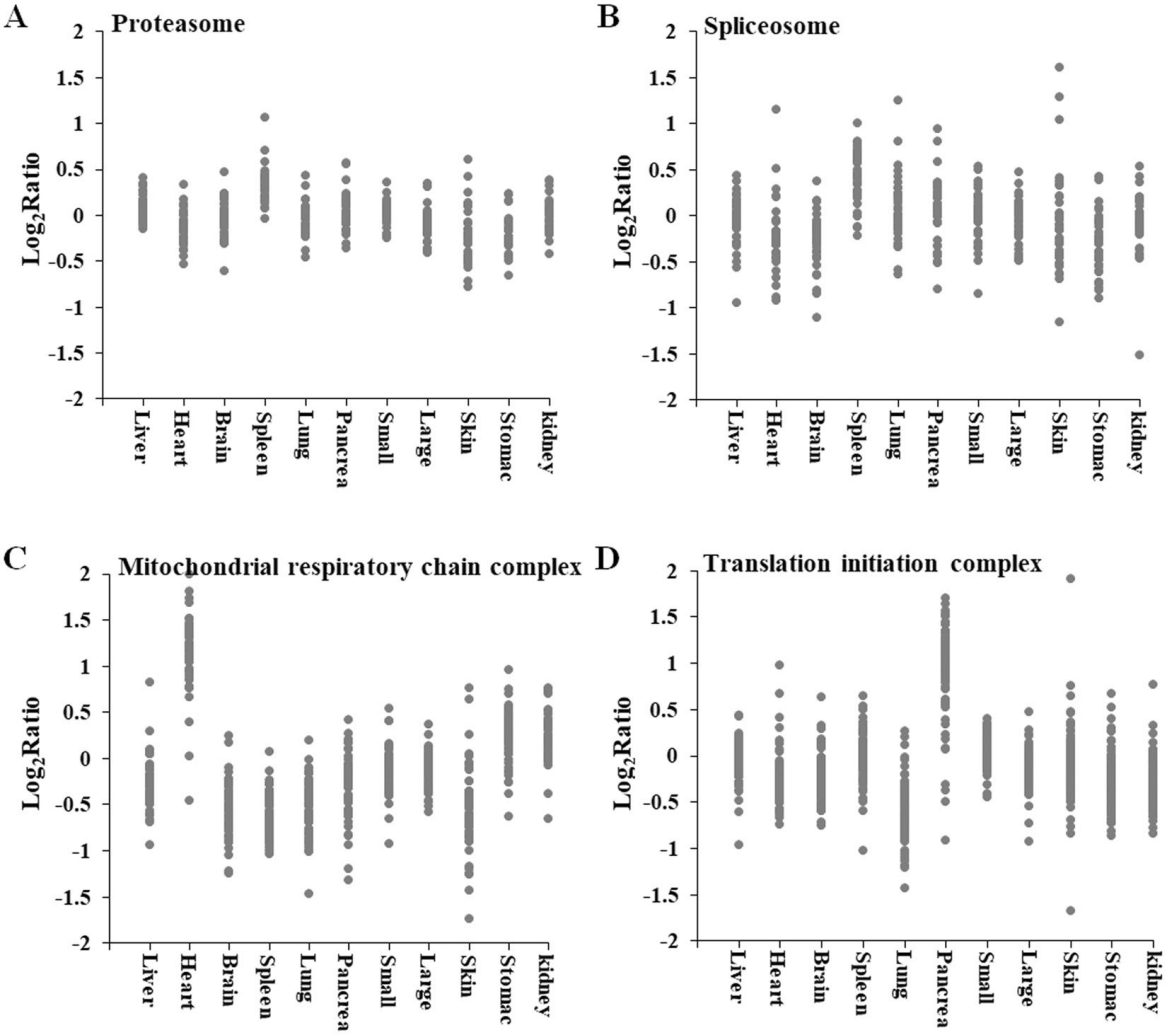
Coregulation of protein complexes in different tissues in the Chinese giant salamander. The graphs indicate the ratios of the subunits of the proteasome (**A**), spliceosome (**B**), mitochondrial respiratory chain complex (**C**), and translation initiation complex (**D**) relative to the internal standard in each of the tissues. The components of molecular complexes are coregulated except for a few outliers in each complex, but the overall level of the complex varies.

This study analyzed the protein levels of members of protein complexes with housekeeping functions, such as ribosomes, proteasomes, spliceosomes and mitochondrial respiratory chain complexes, in eleven CGS tissues. For example, all proteasome subunits had very similar ratios to the internal reference genes in all eleven tissues except for the spleen, where the ratios were higher (Fig. 3A). The spliceosome subunits were also coregulated, but with a higher range of the ratios than proteasome subunits (Fig. 3B). The heart tissue had the highest levels of mitochondrial respiratory chain complexes, followed by the kidney and stomach (Fig. 3C), which may be explained by the high energetic demands of heart for myocardial contraction. The pancreatic tissue had the highest levels of translation initiation complexes, but the lung tissue had the lowest level (Fig. 3D) due to the high translation rates that are needed for the secretory activity of the pancreas. A previous study reported that the protein levels of members of protein complexes, such as ribosomes and proteasomes, were tightly coordinated across 28 mouse tissues^22^. Consistent with these observations, our proteomic data also highlighted the coregulation of protein complexes in eleven CGS tissues, indicating that this pattern is highly conserved across taxa.

### Transcription regulator analysis of tissue-specific proteins

**Figure 4:**
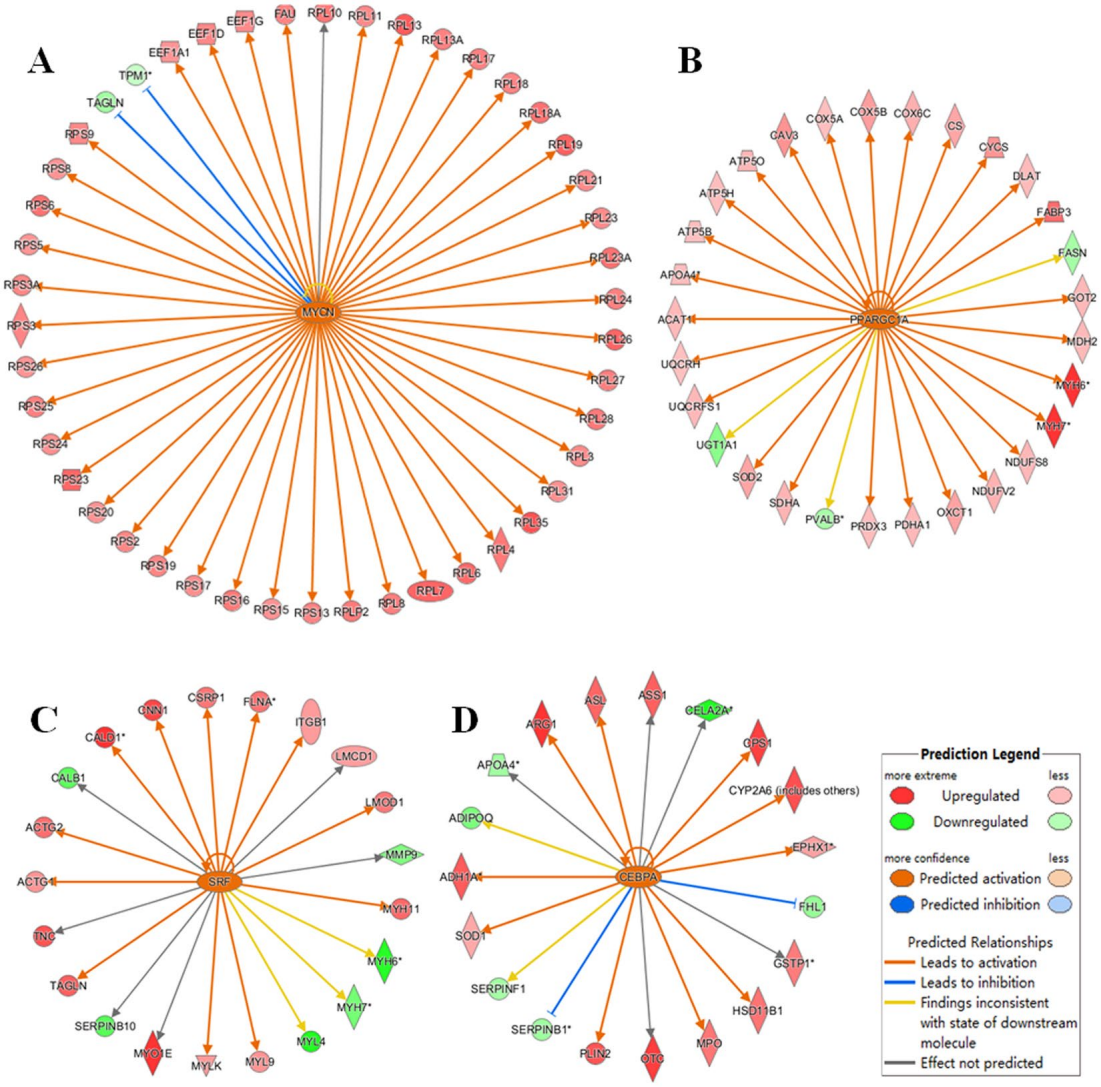
Transcription regulator analysis of tissue-specific proteins by IPA software. (**A**) The activated transcription factor MYCN and its target molecules in the pancreas. (**B**) The activated transcription factor PPARGC1A and its target molecules in the heart. (**C**) The activated transcription factor SRF and its target molecules in the stomach. (**D**) The activated transcription factor CEBPA and its target molecules in the liver.

Transcription factors comprise an important class of regulatory proteins, as they function as on/off switches for gene expression. Ingenuity Pathway Analysis (IPA) software was used to conduct upstream regulator analysis based on overlap *P*-values and activation Z-score. Upstream regulator analysis can identify molecules of interest upstream of the genes in the dataset. The activated transcription regulators and their target genes in each examined CGS tissue are shown in Table S3. The four transcription regulators with the highest predicted activation were MYCN and MYC in the pancreatic tissue and RB1 and PPARGC1A in the heart tissue. MYCN and MYC are members of the MYC family of transcription factors and play a major role in ribosome biogenesis and protein synthesis^23^. In this study, ribosome proteins, as target molecules of MYC, were highly expressed in the pancreas of the CGS (Fig. 4A). Again, our analysis suggests a key role for MYC-regulated ribosome biogenesis in pancreatic tissue that requires high protein translation rates. PGC-1α, encoded by PPARGC1A, is a transcriptional coactivator that regulates the genes involved in energy metabolism and mitochondrial biogenesis^24^. It is also a major factor that regulates muscle fiber type determination. The target proteins of this transcription factor in the dataset were highly expressed in the heart of the CGS (Fig. 4B), suggesting the high-capacity mitochondrial system in the heart. This high capacity is necessary because the constant pumping function requires production and consumption of large amounts of ATP. In addition, the transcription factor SRF was activated in the stomach of the CGS, and its target proteins in the dataset were mainly cytoskeletal proteins that were also highly expressed in the stomach tissue (Fig. 4C). SRF is a downstream mediator of VEGF signaling in endothelial cells and a critical requirement for VEGF-induced angiogenesis^25^. Our analysis highlighted the importance of SRF in stomach protection. The activated transcription factor CEBPA regulates proteins related to metabolism and detoxification, especially cytochrome P450^26^, that were highly expressed in the liver of the CGS (Fig. 4D). These findings indicate that the tissue-activated transcription factors identified here will provide new insights into the regulatory patterns of the different tissues.

### Pathway and bio-functional analysis of tissue-specific proteins

**Figure 5:**
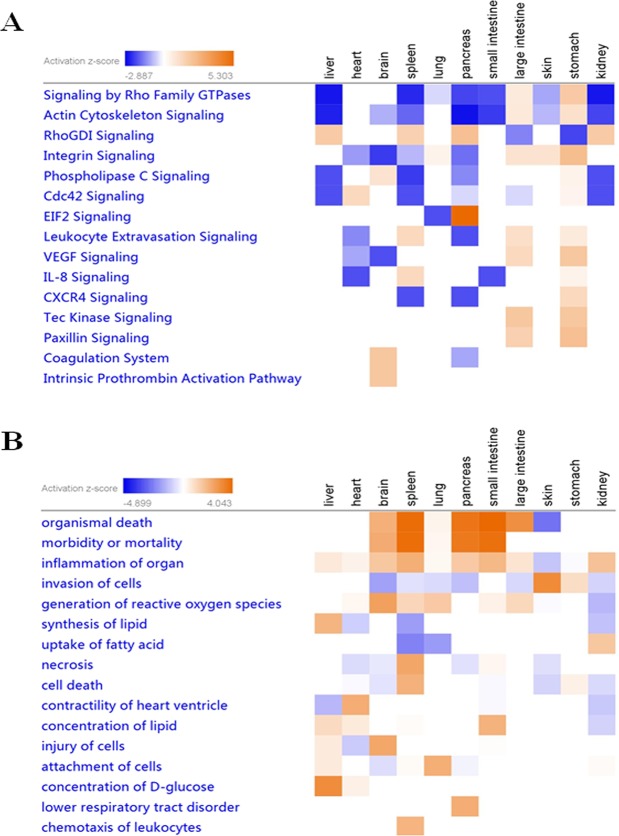
Pathway and bio-functional analysis of tissue-specific proteins by IPA software. (**A**) Pathway analysis of tissue-specific proteins. (**B**) Bio-functional analysis of tissue-specific proteins. Each block diagram is a particular canonical pathway or biofunction. The color orange indicates that the pathway is activated, and the color blue indicates that the pathway is inhibited. Darker colors indicate higher absolute Z-scores.

Beyond these mechanisms of transcriptional regulation, the correlation of tissue-specific proteins with canonical pathways or biofunctions was conducted by core network analysis using IPA software. The activation Z-score is used to predict whether the pathway or biofunction is activated or inhibited in one specific tissue or a few related tissues. The pathway analysis results are summarized in Fig. 5A. The most highly activated canonical pathways in only one tissue or a few related tissues were EIF2 signaling (Z-score 5.303), integrin signaling (Z-score 2.333) and VEGF signaling (Z-score 2). EIF2 signaling, which regulates protein synthesis^27^, was significantly activated in the pancreas, while integrin and VEGF signaling, which regulate cell motility^28^ and angiogenesis^25^, were highly activated in the stomach, again confirming the high translation rates of the pancreas and the rapid peristalsis and wound repair of the stomach. The results of functional analysis demonstrated that many biological processes were predicted to be increased in only one tissue or a few related tissues, such as concentration of D-glucose (Z-score 3.08), contractility of heart ventricle (Z-score 2.219), concentration of lipid (Z-score 2.162), attachment of cells (Z-score 2.157) and chemotaxis of leukocytes (Z-score 2.022) (Fig. 5B). Meanwhile, these processes were also regulated by activated transcription factors, growth factors and cytokines. Concentration of D-glucose in the liver was regulated by activation of the transcription factor CEBPA. Concentration of lipids in the small intestine was regulated by the transcription factor CFTR, which was activated in the small intestine. Increased attachment of cells in the lung was regulated by activation of the growth factor TGFB1. Increased chemotaxis of leukocytes in the spleen was regulated by activation of the cytokine IFNG. These results indicate that the functions of each tissue are largely dependent on tissue-specific proteins.

## Conclusions

We conducted the first quantitative proteome analysis of eleven adult CGS tissues using iTRAQ labeling coupled with LC-MS/MS. Replicated analysis of MS data further improves the accuracy and reliability of proteomic data. The number of differentially regulated proteins in each tissue is similar to that obtained in previous studies that included multiple tissues^22,29^. Furthermore, we provided expression patterns of thousands of proteins across eleven CGS tissues and identified 922 tissue-specific proteins. Our proteomic data also highlighted the coregulation of protein complexes in eleven CGS tissues. Compared with other tissues, EIF2 signaling and transcription regulators MYCN and MYC were significantly activated in the pancreas, integrin and VEGF signaling were highly activated in the stomach, and RB1 and PPARGC1A were significantly activated in the heart. In total, our initial quantitative proteome map of this tailed amphibian provides a useful resource for others to seek proteins of interest that are differentially regulated between tissues. Our data, as an initial atlas of protein expression in an amphibian species, will be useful to molecular research on the giant salamander.

## Materials and Methods

### Animals and sample preparation

Three adult CGSs, each weighing approximately 2 kg, were obtained from an artificial breeding base belonging to Chongqing Kui Xu Biotechnology Incorporated Company. The giant salamanders were reared in aerated, tap-water-supplied tanks at 20 °C and fed diced bighead carp for 2 weeks prior to experiment. The animals were heavily anesthetized with the anesthetic MS-222 and sacrificed by dissection before sample collection. Multiple tissues (skin, spleen, lung, heart, kidney, liver, pancreas, stomach, small intestine, large intestine and brain) were collected, flash frozen in liquid nitrogen and stored at −80 °C until use. All animal experiments in this study were performed in strict accordance with the guidelines of the Animal Care and Use Committee of Henan Normal University and were approved by the Animal Care and Ethics Committee of Henan Normal University.

### Protein extraction and iTRAQ labeling

Protein extraction was performed using a procedure described previously^30^. Briefly, the frozen tissue was homogenized, ultrasonically disrupted and centrifuged, and the supernatant was collected. The concentration of protein was measured using the BCA method. Each protein sample was first denatured, reduced, alkylated, digested and subjected to OD280 peptide quantitation according to the method previously described^31^. Then, the tryptic peptides of each sample were labeled according to the instructions of the iTRAQ Reagent 8PLEX Multiplex Kit (AB SCIEX). The 113, 114, 115, 116, 117, 118, 119 and 121 tags in one iTRAQ Reagent 8PLEX Multiplex Kit (AB SCIEX) were utilized to label the tryptic peptides from liver, heart, brain, spleen, lung, pancreas, small intestine and large intestine samples, respectively. Meanwhile, 117, 118, 119 and 121 in another iTRAQ Reagent 8PLEX Multiplex Kit were utilized to label the tryptic peptides from liver, skin, stomach, and kidney samples, respectively. The liver sample served as a common internal reference. Subsequently, the iTRAQ labeled peptides in each Reagent Kit were pooled and vacuum dried.

### LC-MS/MS analysis for protein identification

Strong cation exchange chromatography and protein identification by mass spectrometry were performed according to the method previously described^30,32^. A total of 33 fractions were collected over the gradient, but some were pooled to give a final total of 15 fractions based on the SCX chromatogram. The fractions were desalted using a PepClean C-18 spin column (Sigma, USA)^33^ and dried with a vacuum centrifugal concentrator. Then, each fraction was separated by capillary high-performance liquid chromatography on a Thermo Scientific EASY-nLC 1000 system (Thermo Finnigan, USA)^34^, and the separated samples were analyzed with a Q Exactive mass spectrometer (Thermo Finnigan, USA)^35^. The parameters of MS were set as follows: positive ion detection mode, precursor ion scan range of 300–1800 m/z, MS resolution of 70,000 at m/z 200, AGC target of 3e6, MS maximum IT of 10 ms, number of scan ranges of 1, and dynamic exclusion of 40.0 s. The mass-charge ratios of peptide fragments and peptides were collected using the following settings: collecting 10 fragments spectra after a full MS2 scan, MS2 activation type of HCD, isolation window of 2 m/z, MS2 resolution of 17,500 at m/z 200, microscan count of 1, MS2 maximum IT of 60 ms, normalized collision energy of 30 eV, and underfill ratio of 0.1%.

### Data analysis

For peptide data analysis, raw data were processed using Proteome Discoverer 1.4 software (Thermo Scientific) and searched against the predicted protein database from the CGS transcriptome database (SRA accession number: SRP092015) using Mascot 2.2 (Matrix Science, London, UK). The search parameters were set as follows: MS/MS ion search, trypsin as the digestion enzyme with allowance for a maximum of two missed cleavage points, carbamidomethyl (C) and iTRAQ 8-plex modification (K and N-terminus) as a fixed modification, oxidation (M) as a variable modification, peptide mass tolerance of ±20 ppm, fragment mass tolerance of 0.1 Da, and unrestricted protein mass. The mass spectrometry data have been provided to the ProteomeXchange Consortium via the partner repository PRIDE^36^ with the dataset identifier PXD005648.

The following filters were used in this study: a false discovery rate (FDR) ≤ 0.01 and at least 2 unique peptides per protein^37^. We first calculated the ratio value by comparing the expression level of each protein in liver tissue to those in the other ten tissues, since the liver sample served as a common internal reference in the two iTRAQ Reagent 8PLEX experiments. Then, the data from the eleven tissues were integrated, and the average ratio value of each protein in all tissues was used as an internal reference. Subsequently, the relative expression quantity was calculated for each protein to facilitate the identification of proteins with low absolute expression values but with significant expression changes in specific tissues. Based on relative expression quantity, proteins with ratio values > 2 were considered to be highly expressed in specific tissues^38^.

### Western blot analysis

Western blot analysis was performed according to Towbin’s method^39^. Antibodies against salamander proteins were not available; therefore, mammalian antibodies were used in our study. Briefly, proteins were separated by 12% SDS-PAGE and transferred to a nitrocellulose membrane. The membranes were incubated with rabbit antibodies (anti-ANPEP, anti-ALDH6A1, anti-GOT2, anti-ADH1, anti-HMOX1 and anti-SOD1) overnight at 4 °C. Then, the membrane was incubated with horseradish peroxidase (HRP)-conjugated goat anti-rabbit IgG secondary antibodies (Sigma, 1:2500). Finally, the protein bands were visualized with Amersham enhanced chemiluminescence (ECL) substrates. Exposure imaging was performed on the ImageQuant LAS 4000 Chemiluminescence Imager (GE Healthcare), and the band density was measured using ImageQuant TL software (GE Healthcare). β-Actin (Sigma, 1:1000) served as an internal reference.

### Bioinformatic analysis

Functional annotation and pathway assignment were carried out based on the annotation results of the CGS transcriptome^6^. WEGO was used to perform GO functional classification^40^. Co-expression modules were constructed by using weighted gene co-expression network analysis (WGCNA)^41^. DAVID analysis was applied to conduct functional and pathway enrichment analysis according to a modified Fisher’s exact test in combination with the FDR method as described in detail elsewhere^42^. Principal component analysis (PCA) was conducted using IBM SPSS Statistics 19.0. Signal-peptide prediction of the identified proteins was performed using SignalP 4.0^43^.

The tissue-specific expressed proteins were analyzed by Ingenuity Pathway Analysis (IPA) version 9.0 (Redwood City, CA, http://www.ingenuity.com) software for transcription regulators, canonical pathways and biological functions^44^. The core analysis and comparison analyses of proteins were conducted successively to generate a heatmap for all examined tissues^45^. The activation Z-score was used to predict whether the downstream process was increased or decreased. An activation Z-score > 2 demonstrates that a regulator is highly activated or a process is significantly increased in a specific tissue in comparison to the internal standard for all tissues.

## Supplementary information

Supplementary information

Table S1

Table S2

Table S3

## Data Availability

The mass spectrometry proteomic data have been provided to the ProteomeXchange Consortium via the partner repository PRIDE with the dataset identifier PXD005648.
