# Supplementary material for: Proteomic analysis of eleven tissues in the Chinese giant salamander (Andrias davidianus)
Source: Sci Rep. 2019 Nov 11;9:16415. doi: 10.1038/s41598-019-50909-3 (PMC6848178; doi:10.1038/s41598-019-50909-3)
Supplement: Supplementary file 1 — Supplementary information [file 41598_2019_50909_MOESM1_ESM.pdf]

Proteomic analysis of eleven tissues in the Chinese giant salamander (*Andrias davidianus*)

Xiaofang Geng[1,2], Jianlin Guo[1], Xiayan Zang[1], Cuifang Chang[1], Haitao Shang[4], Hong Wei[3,*], Cunshuan Xu[1,*]

[1] State Key Laboratory Cultivation Base for Cell Differentiation Regulation, College of Life Science, Henan Normal University, Xinxiang, China

[2] Henan Key Laboratory of immunology and targeted therapy, Henan Collaborative Innovation Center of Molecular Diagnosis and Laboratory Medicine, School of Laboratory Medicine, Xinxiang Medical University, Xinxiang, China

[3] The Engineering Technology Research Center for Germ-free and Genome-editing animal, Key Laboratory of Agricultural Animal Genetics, Breeding, and Reproduction of the Ministry of Education & Key Laboratory of Swine Genetics and Breeding of Ministry of Agriculture and Rural Affairs, The Cooperative Innovation Center for Sustainable Pig Production, Huazhong Agricultural University, Wuhan, China.

[4] Department of Laboratory Animal Science, College of Basic Medical Sciences, Third Military Medical University, Chongqing, China

*Correspondence should be addressed to Cunshuan Xu (cellkeylab@126.com) and Hong Wei (weihong63528@163.com).

Supplemental Figures

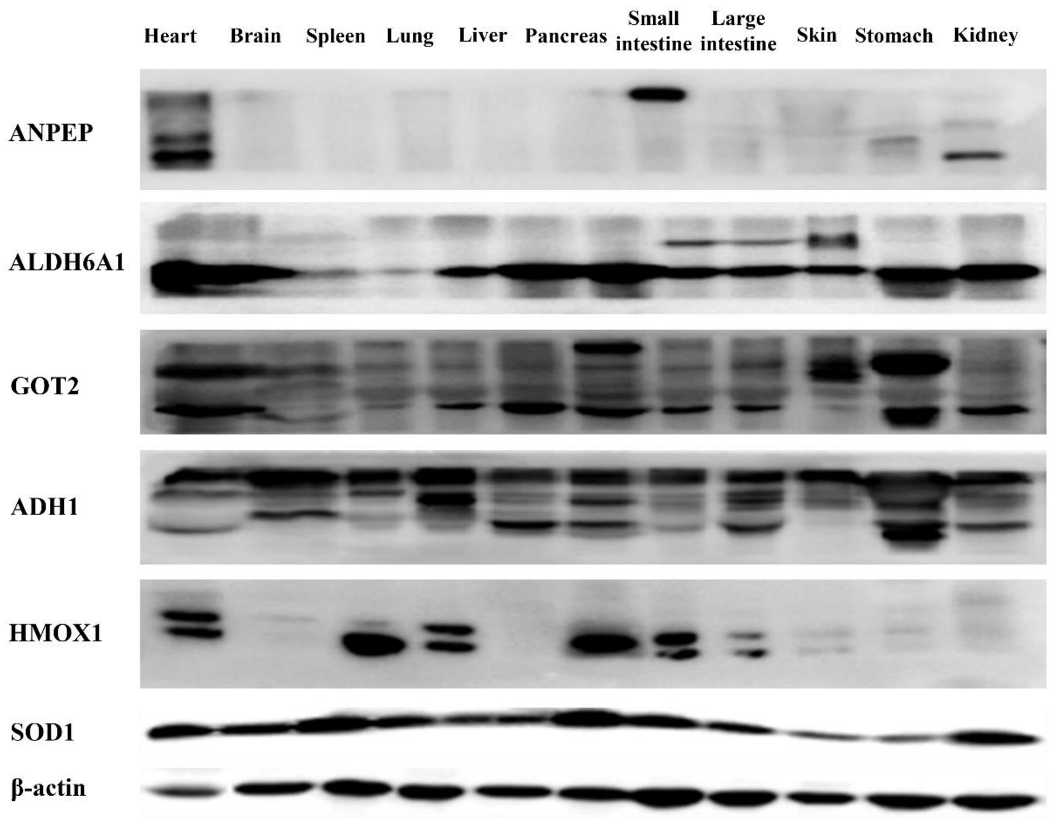

Figure S1. The original scans for all the western blots.

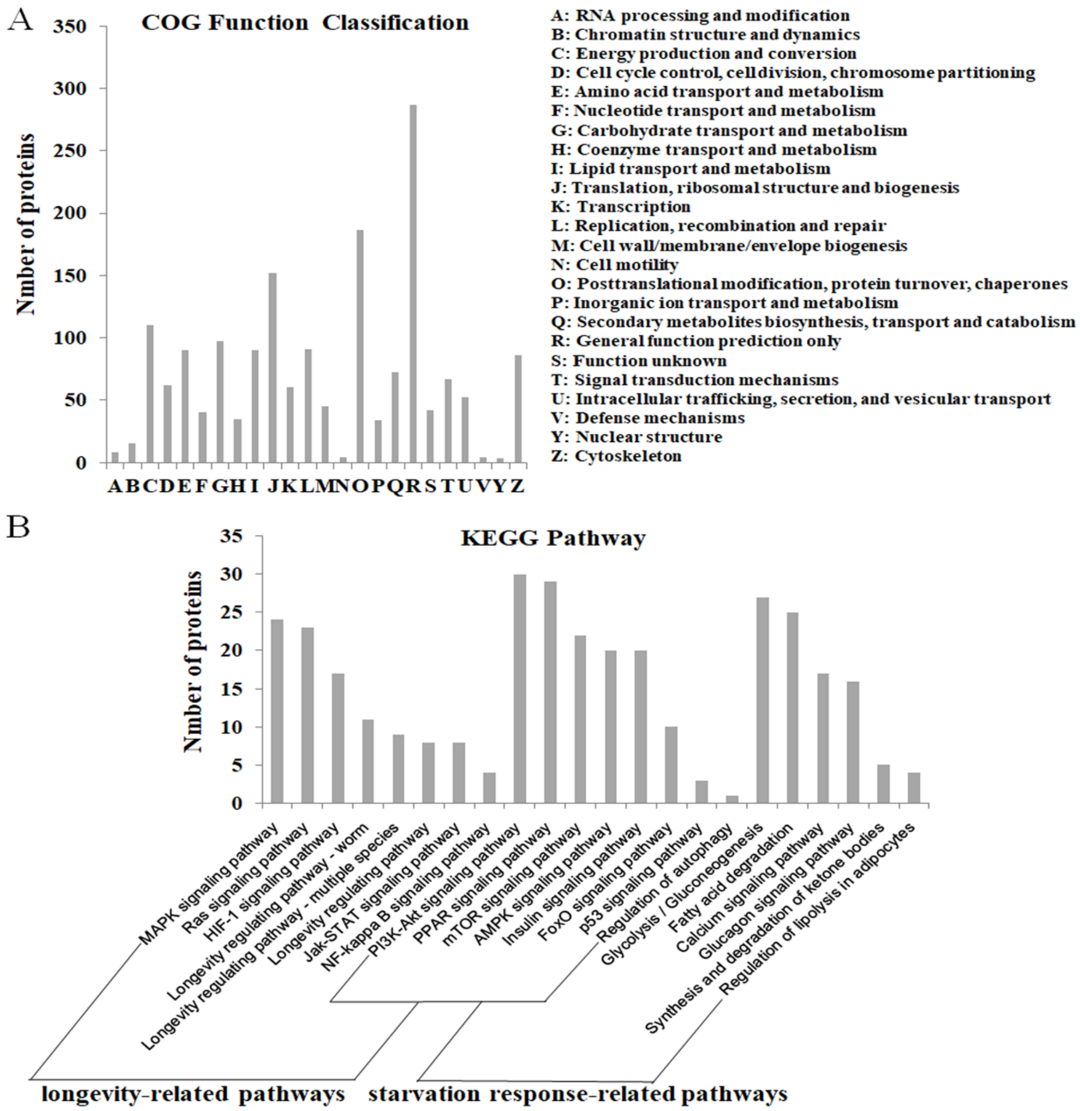

Figure S2. COG and KEGG pathway classification of tissue proteomes of Chinese giant salamander. (A) Histogram of cluster of orthologous groups (COG) classification. (B) Categories classified by KEGG pathway.

Supplemental Tables

Table S1. The identified proteins, tissue-specific proteins and secreted proteins of Chinese giant salamander.

Table S2. Lists of proteins for co-expression modules.

Table S3. The activated transcription regulators and its target genes in the dataset of Chinese giant salamander.
